# Lightweight multi-scale network for small object detection

**DOI:** 10.7717/peerj-cs.1145

**Published:** 2022-11-08

**Authors:** Li Li, Bingxue Li, Hongjuan Zhou

**Affiliations:** 1 School of Information and Electrical Engineering, Hebei University of Engineering, Handan, China; 2 China Water Resources & Hydropower Engineering Bohai Consultancy Co. Ltd, Tianjin, China

**Keywords:** Small object detection, Multi-scale feature fusion, Receptive field enhancement, Channel attention

## Abstract

Small object detection is widely used in the real world. Detecting small objects in complex scenes is extremely difficult as they appear with low resolution. At present, many studies have made significant progress in improving the detection accuracy of small objects. However, some of them cannot balance the detection speed and accuracy well. To solve the above problems, a lightweight multi-scale network (LMSN) was proposed to exploit the multi-scale information in this article. Firstly, it explicitly modeled semantic information interactions at every scale via a multi-scale feature fusion unit. Secondly, the feature extraction capability of the network was intensified by a lightweight receptive field enhancement module. Finally, an efficient channel attention module was employed to enhance the feature representation capability. To validate our proposed network, we implemented extensive experiments on two benchmark datasets. The mAP of LMSN achieved 75.76% and 89.32% on PASCAL VOC and RSOD datasets, respectively, which is 5.79% and 11.14% higher than MobileNetv2-SSD. Notably, its inference speed was up to 61 FPS and 64 FPS, respectively. The experimental results confirm the validity of LMSN for small object detection.

## Introduction

Object detection (Zaidi et al., 2022) plays an important role in the field of computer vision. It aims to accurately identify the objects in input images and precisely locate them. In recent years, deep learning-based methods, such as Faster R-CNN (Ren et al., 2015), YOLO (Redmon et al., 2016) and SSD (Liu et al., 2016) have stimulated significant progress in object detection. Those methods perform well for large, high-resolution and clear objects. However, they usually cannot accurately detect small targets, since rich feature representations are hard to learn from low-resolution small objects. Small object detection is widely common in real life applications, for instance, intelligent monitoring (Kumar, Punitha et al., 2020), medical treatment (Ganatra, 2021) and autonomous driving (Feng et al., 2020). However, it is difficult to detect small objects in complex real-world scenes due to the low resolution and heavy occlusion. Therefore, quickly and effectively detecting small objects has become an urgent challenge.

In recent years, a series of deep learning-based methods were proposed to detect small objects. Pang et al. (2019) proposed a lightweight featured image pyramid network that combined feature attention and forward fusion to detect small objects. Ma & Zhou (2020) introduced an attention module to effectively extract small object features and adopted feature fusion to accurately regress the small target positions. Qu et al. (2020) fused shallow feature maps and deep feature maps to strengthen the semantic representation ability of shallow feature maps. At the same time, dilated convolution was adopted to enhance receptive fields of feature maps. Zhai et al. (2020) applied DenseNet to extract features and designed a novel feature fusion mechanism, which increased the detection performance for small object detection. A feature-aligned pyramid network was proposed by Huang et al. (2021), the network integrated a feature alignment module and a feature selection module to improve model performance. Qian et al. (2020) proposed a distributional ranking loss that tackled the problem of class imbalance for small objects. According to the results, the method greatly improved the small object detection performance. Zhang & Jiang (2022) constructed a recursive inverse path from high-level to low-level, which included a deep feature enhancement module, an up-sampling feature enhancement module and an adaptive feature fusion module. Fan et al. (2020) integrated Faster R-CNN, Tiny Face and CNN for helmet wearing detection, which improved the precision and recall of existing methods in complex scenes.

Although these methods achieved promising results, there are still challenges in small object detection. On the one hand, due to the lack of semantic information in low-level feature maps, small objects are not detected accurately. On the other hand, the small objects have few pixels and low resolution, which leads to insufficient features for detection. In addition, the above methods focus on improving small object detection accuracy at the expense of detection speed, which cannot well balance accuracy and speed.

In this article, we propose a lightweight multi-scale network (LMSN) with high-precision and high-speed to solve the above problems. Different from the existing methods, LMSN is equipped with three innovative design modules, namely multi-scale feature fusion (MSFF), lightweight receptive field enhancement (LRFE) and efficient channel attention (ECA). MSFF is a feature fusion module, which is designed to merge low-level detail features and high-level semantic features to enhance the semantic expression of small objects in low-level feature maps. At the same time, LRFE is employed in the process of feature fusion to enhance the small object feature extraction ability by simulating human vision receptive fields. Finally, ECA is introduced to pay more attention to the significant information and enhance the feature representation ability. The experimental results demonatrate that the LMSN significantly improves the small object detection accuracy while meeting the real-time detection requirement.

The main contributions of this work can be summarized as follows: (1) A novel LMSN was proposed for small object detection, which facilitates the robust and quick learning of small object detection in complex real-world scenes. (2) Three innovative modules were well-designed to address the key issue that small objects are difficult to detect. At the same time, the network achieves a good balance between accuracy and speed. (3) The proposed LMSN established comparable performance in small object detection on two different benchmark datasets. Experimental results show that LMSN greatly improves detection accuracy with competitive inference speed.

The rest of this article is organized as follows. “Related work” describes a review of the literature. “Proposed Method” introduces our proposed LMSN architecture in detail. “Experiments and Results” presents the experimental results and their analysis. Finally, “Conclusion” presents the conclusions in this article.

## Related Work

In this section, we briefly review related work on object detection methods, feature fusion methods, receptive field enhancement methods, and attention mechanism.

### Object detection methods

According to the feature extraction method, the current object detection methods can be divided into two categories: traditional object detection methods and deep learning-based object detection methods. In the early years, traditional object detection methods (Viola & Jones, 2001; Dalal & Triggs, 2005; Felzenszwalb, McAllester & Ramanan, 2008) mainly rely on hand-crafted features. The extracted features are relatively single, and the precision of object detection in complex scenes is low, which limits the wide application of such methods. With the development of artificial intelligence, deep learning methods are increasingly used in classification and detection tasks (Yu et al., 2022). Deep learning-based object detection methods extract features through powerful deep convolutional neural networks, which can generally be divided into two classes. The first one is two-stage method based on the regional proposal, such as R-CNN (Girshick et al., 2014), Fast R-CNN (Girshick, 2015), Faster R-CNN, R-FCN (Dai et al., 2016), Mask R-CNN (He et al., 2017) and so on. Although this kind of method has excellent detection effect for small-scale objects, the calculation process is complex. Therefore, the detection speed of two-stage methods is slow, which is not suited for real-time applications. The other one is one-stage method based on regression, such as YOLO series (Redmon & Farhadi, 2017; Redmon & Farhadi, 2018; Bochkovskiy, Wang & Liao, 2020), SSD, RetinaNet (Lin et al., 2017b), EfficientDet (Tan, Pang & Le, 2020) and so on. This kind of method uses end-to-end approach to detect objects. The detection speed is faster compared to the two-stage methods, but the detection effect is not very satisfactory.

To trade off the inference speed and detection accuracy of current object detection models, the MobileNetv2-SSD algorithm (Huang et al., 2022a) came into being, which adopts MobileNetv2 as the backbone network to extract features. MobileNetv2-SSD accomplishes a satisfactory balance by taking into account speed and accuracy, which has been widely concerned and broadly used in object detection. Despite the MobileNetv2-SSD algorithm achieving superior performance, there is always room for more accurate small object detection. A multi-scale network model was proposed by Chen et al. (2019) for small target detection, which can learn rich small object features. Sun et al. (2021) improved the small object detection performance by enhancing detection features. Wu et al. (2021) combined the idea of feature pyramid network to merge feature layers that contain rich semantic information. The method improved the model performance for detecting small objects. The above methods improve the model detection performance for small-scale objects at the expense of detection speed, which is not conducive to real-time application in real life. This article adds three effective modules based on MobileNetv2-SSD to construct a lightweight and efficient multi-scale network. The network not only ensures a good balance of accuracy and speed but also greatly improves the detection performance of small objects.

### Feature fusion

The current object detection methods can accurately detect large objects in complex scenes, but they are not suitable for small object detection. Feature fusion can effectively solve this problem and enhance the model detection performance. A large number of previous works have proposed many feature fusion methods. Lin et al. (2017a) constructed a top-down structure with lateral connections, called Feature Pyramid Network (FPN). The structure utilized upsampling and lateral connections to generate feature maps with stronger semantic information. Liu et al. (2018) added a bottom-up path augmentation with shortcut connections based on FPN. Path Aggregation Network (PANet) was proposed to further enhance the localization ability of the network. Guo et al. (2020) proposed an Augmented FPN (AugFPN), which consisted of consistent selection, residual feature augmentation, and soft ROI selection. The AugFPN effectively solved the problem of semantic gaps and information loss in FPN. Tan, Pang & Le (2020) designed a weighted bi-directional feature pyramid network (BiFPN), which utilized cross-scale connections and weighted feature fusion to learn the importance of different features. The method greatly improved detection efficiency. Jiang et al. (2022) developed a novel generalized-FPN (GFPN) structure composed of queen-fusion style pathway and skip-layer connection, which greatly improved the model accuracy.

Based on the above research, many feature fusion methods have been applied in object detection. Leng & Liu (2019) used bidirectionally transmitted feature information to fusion feature maps from different output layers, which enhanced the network performance. Fan et al. (2019) proposed a feature fusion block consisting of a feature aggregation block and a dense feature pyramid, which significantly improved model accuracy and maintained a close detection speed. A cross-scale feature fusion structure was designed by Cheng et al. (2020a), the structure combined feature pyramid network and squeeze-and-excitation block. Shahin & Almotairi (2021) used an additive fusion function to fusion the spectral saliency features map and spatial features map. The results on the self-built desert building dataset show that the architecture can effectively improve detection performance. The existing feature fusion methods have complex network structures and long detection pipelines, which significantly reduce the detection speed. Different from the above methods, our proposed feature fusion module achieves excellent detection performance, which simplifies the fusion structure and reduces network complexity. The feature pyramid network newly generated by the feature fusion module effectively detects small-scale targets while ensuring the detection speed.

### Receptive field enhancement

The low-level feature maps extracted by backbone network have a small receptive field, which is unfavorable for small target feature recognition. Many studies solve this problem by expanding the receptive field of feature maps. A series of methods have been proposed to enlarge the receptive field. He et al. (2015) adopted spatial pyramid pooling to arbitrarily scale the input images. The method obtained a fixed-length representation, which avoided repeated convolution calculations and greatly speeded up detection. Zhao et al. (2017) designed a pyramid pooling module, which effectively increased the receptive field and enhanced the utilization of global information. The module used four different scale pooling operations to obtain multi-scale feature maps and formed the final feature representation through channel splicing. Chen et al. (2017) proposed Atrous Spatial Pyramid Pooling (ASPP) that employed atrous convolution with multiple atrous rates to capture multi-scale semantics. The method enlarged the receptive field without losing resolution. Inspired by the structure of Receptive Fields (RFs) in the human visual system, Liu, Huang et al. (2018) proposed a novel Receptive Field Block (RFB). This design generated higher resolution feature maps, which captured more contextual information. Li et al. (2019) presented a Trident Network that utilized multi-branch structures with different receptive fields to detect objects. Zhang et al. (2020) added a multi-scale atrous convolution module to enlarge the receptive field of feature layers and enhance the learning ability of the network. Liu et al. (2021) designed mixed dilated convolution with different sampling rates, which expanded the receptive field and improved the small object detection performance. Huang et al. (2022b) designed a novel Parallel-insight Convolution layer to extract information from different domains, which was integrated with a Spatial-Temporal Dual-Attention unit to extract high-quality global spatial–temporal features. This method effectively improved cross-view gait recognition. Wang et al. (2021) proposed a temporal dilated dense prediction block consisting of Spatial Global Pooling, Channel Compression, and Temporal Dilated Dense Prediction Layer. The method achieved excellent performance in video action recognition while maintaining low computational cost. Inspired by the above research, we design a lightweight receptive field enhancement module. The module refines the detection branch, which uses parallel convolutions and serial convolutions to greatly reduce computation. At the same time, dilated convolutions with different dilation rates are applied to enlarge the receptive field of the feature layers. In addition, the receptive field enhancement module is added to the feature fusion module to obtain features with higher resolution, which further enhances the detection performance.

### Attention mechanism

Recently, attention mechanism has been largely exploited in object detection methods. In the process of small object detection, the attention mechanism can suppress irrelevant background information and strengthen the key features. More and more attention is paid to the development of attention mechanisms. Hu, Shen & Sun (2018) designed the Squeeze-and-Excitation (SE) block to recalibrate channel feature representation by learning the interrelationships between channels. The structure significantly enhanced the network performance with only a small increase in computational cost. Woo et al. (2018) developed a Convolutional Block Attention Module (CBAM) to adaptively refine features in both channel and spatial dimensions. Cao et al. (2019) proposed a global context (GC) block, which effectively modeled the global context. Wang et al. (2020) designed an Efficient Channel Attention (ECA) module that adaptively learned channel features using a local channel interaction strategy.

Based on the above studies, attention mechanisms are widely used in object detection methods. Gao, Cai & Ming (2020) developed a residual module with an efficient channel attention mechanism, called ECA-ResNet. The architecture enhanced the connection between each feature map through global average pooling and local cross-channel interaction operation. Li et al. (2020) designed new attention units to adaptively implement attention mechanism across channels, spaces, and domains. Lu et al. (2021) presented a novel attention module with two paths to restrain background interference information and highlight important feature information. Dong et al. (2022) developed a Shuffle Polarized Self-Attention (SPSA) to generate more discriminative feature representations adaptively in the channel and spatial dimensions. The detector combined with SPSA achieved excellent detection results in the wheat ear detection task. Although our proposed feature fusion module combines the features of shallow and deep layers, the correlation between each feature layer is weak. Therefore, we introduce an efficient channel attention module after the feature pyramid newly generated by the feature fusion module. The module learns feature information between feature layers by assigning different weights. In addition, the scheme of combining feature fusion and attention mechanism not only strengthens the network’s attention to key features but also enhances the feature expression ability.

## Proposed Method

In this section, we first illustrate the network architecture of LMSN. Then, we describe the proposed MSFF module, LRFE module and ECA module in detail.

### The network architecture

The MobileNetv2-SSD is an improved method based on SSD, using MobileNetv2 instead of VGG16 as backbone network. The MobileNetv2-SSD object detection method can greatly cut down the amount of calculation and speed up the inference speed. But for small object detection, it is not satisfactory. The method selects six feature layers of different scales from the extracted feature layers for prediction. These feature layers are independent of each other so that the detailed features of low-level feature maps and the semantic information of high-level feature maps are not fully utilized. This shortcoming leads to the problem of missed detection and false detection of small objects. In addition, the method only predicts objects separately on each feature layer. The connection of these feature layers is weak and information interaction is incomplete. These problems cause the key features to be easily interfered by background information, which makes the network ineffective for small target detection.

**Figure 1:**
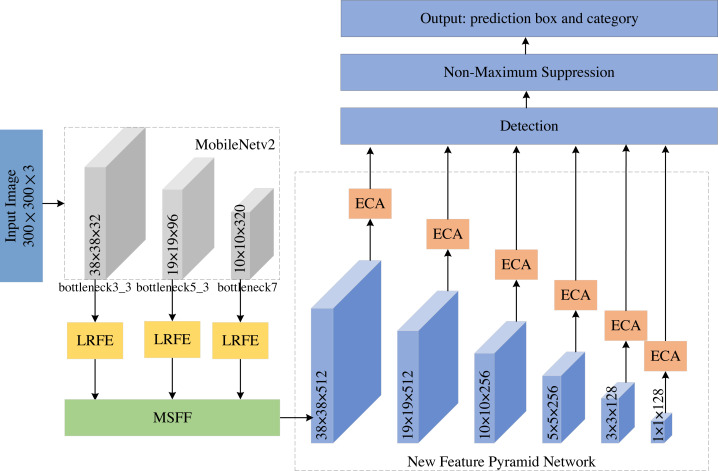
The network architecture of LMSN.

To improve the detection performance of small objects, we propose an improved MobileNetv2-SSD object detection method, named LMSN. As shown in Fig. 1, the LMSN mainly includes three modules: MSFF module, LRFE module and ECA module. (1) Firstly, MSFF is developed to fuse feature layers of different scales, which introduces richer semantic expressions into feature maps with more detailed information. Then a new multi-scale feature pyramid network is generated to detect different objects. (2) Next, we add LRFE in feature extraction process to dilate the receptive field and enhance the feature extraction capability. (3) Finally, ECA is adopted to strengthen the association between each feature map. The ECA can suppress the irrelevant background information and emphasize the key information. The LMSN effectively increases the detection performance of small-scale objects without reducing the inference speed.

The overall network framework of LMSN is as follows: The input image size of LMSN is 300 × 300. We first use MobileNetv2 as the backbone network to extract features, which generates three effective feature layers bottleneck3_3, bottleneck5_3, and bottleneck7. Specifically, they are the feature of the third convolution of bottleneck3, the feature of the third convolution of bottleneck5, and the feature of the convolution of bottleneck7, respectively. These feature layers are input to the LRFE module to increase the receptive field. Then, the high-level feature and low-level feature are fused through the MSFF module to obtain a new feature layer containing richer information. Five convolution operations with stride 2 are performed on the new feature layer to generate a new feature pyramid network, which consists of six feature layers of different scales. Next, we use the ECA module to suppress the irrelevant background information of these feature layers, so that the network pays more attention to key features. Finally, the feature maps output by the New Feature Pyramid Network from high to low are 38 × 38, 19 × 19, 10 × 10, 5 × 5, 3 × 3, and 1 × 1. The feature maps with sizes of 38 × 38 and 19 × 19 are high-level feature maps, which are large and suitable for detecting small objects. The feature maps with sizes of 10 × 10, 5 × 5, 3 × 3, and 1 × 1 are low-level feature maps, which is small and suitable for detecting large objects.

During the detection process, the effective feature layers are divided into grids, and corresponding default boxes of different scales and aspect ratios are generated on each grid. Classification and regression are performed for each feature layer to predict the category and location of the object. The regression branch continuously adjusts the prior boxes to approximate the ground truth boxes of the objects. The probability of classification is calculated by the softmax function.

The calculation method of the default boxes is as follows: The scales of the default boxes generated on the feature map follow the linear increasing rule: As the size of the feature map decreases, the scale of the default box increases linearly, as shown in Eq. (1).

(1) \begin{eqnarray*}{s}_{k}={s}_{min}+ \frac{{s}_{max}-{s}_{min}}{{s}_{min}} (k-1),k\in [1,m]\end{eqnarray*}

where *s*_*min*_ = 0.2, *s*_*max*_ = 0.9, *m* is the number of feature maps.

The aspect ratios of each default box are set to ${a}_{r}\in \{ 1,2,3, \frac{1}{2} , \frac{1}{3} \} $. In this way, we can calculate the width (${w}_{k}^{a}={s}_{k}\sqrt{{a}_{r}}$) and height (${h}_{k}^{a}={s}_{k}/\sqrt{{a}_{r}}$) of each default box. When the aspect ratio is 1, we also add a default box whose scale is ${s}_{k}^{{}^{{^{\prime}}}}=\sqrt{{s}_{k}{s}_{k+1}}$, resulting in 6 default boxes per grid. By combining the default boxes with different scales and aspect ratios, we can easily detect objects of different scales.

After the detection, the repeated prediction boxes are removed by the Non-Maximum Suppression (NMS) algorithm to obtain the final detection results. The NMS algorithm ranks the prediction boxes of each category from high to low in terms of classification confidence. In a certain category, the box with the highest confidence is selected first. Then the intersection over union (IOU) of this box with the rest of the boxes is calculated. When the IOU is higher than the set threshold, the corresponding box is deleted.

### Multi-scale feature fusion module

The MobileNetv2-SSD method utilizes multi-scale feature layers to classify and predict objects of various scales and shapes. The large-scale low-level feature maps are suitable for detecting small-scale objects. The small-scale high-level feature maps are mainly used to detect large-scale objects. Although the large-scale low-level feature maps have rich detailed information and high resolution, the receptive field is small and the semantic expression ability is weak. The small-scale high-level feature maps have large receptive field and rich semantic representation, but the detailed information representation ability is weak and the resolution is small. In addition, these feature maps are independent of each other and feature information is relatively single. Therefore, the detection precision of MobileNetv2-SSD is lower for small object detection.

**Figure 2:**
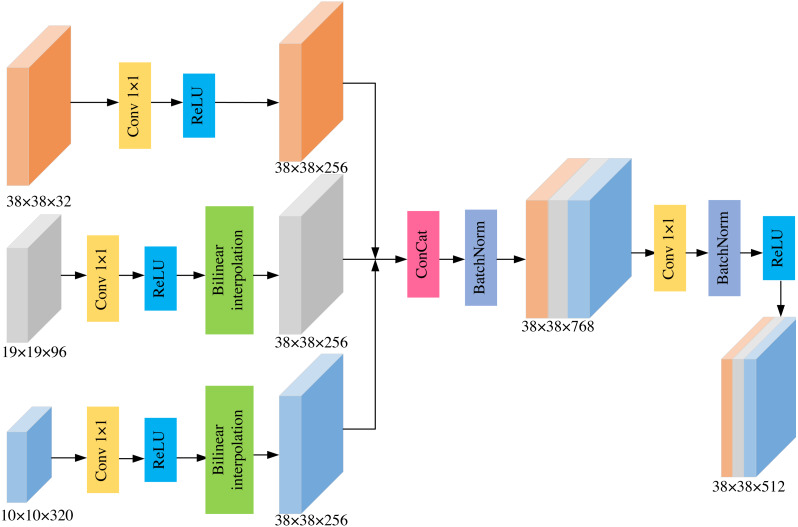
The structure of MSFF module.

To solve the above-mentioned problem, we propose a lightweight and efficient MSFF module by combining the idea of FPN. The MSFF module fuses the detailed features of the large-scale low-level feature maps and the semantic information of small-scale high-level feature maps. The module finally obtains the low-level feature maps containing richer semantic feature representation, which effectively solves the problem of unsatisfactory detection performance for small-scale targets. The structure of MSFF is shown in Fig. 2.

MobileNetv2-SSD uses six different scales of feature maps for object detection. The corresponding feature sizes are 19 × 19, 10 × 10, 5 × 5, 3 × 3, 2 × 2, and 1 × 1. We think that feature maps whose spatial size is smaller than 5 × 5 have little semantic information to merge. If these feature layers are merged with large-scale feature layers, it will only increase the computational of the model and decrease the detection speed. So we do not fuse these feature maps. In addition, the feature map with the largest scale is 19 × 19 of the bottleneck5_3 layer, which is still insufficient for the detection of low-resolution small targets. The feature layer with a scale size of 75×75 also has no benefit to final performance. So we incorporate the feature map with a scale size of 38 × 38 into MSFF.

The process of MSFF module is as follows. (1) Firstly, 1 × 1 convolution is used for each feature map to reduce the channel dimension, so that the channel dimension in each feature map is consistent. (2) Next, we set the size of bottleneck3_3′s feature layer as the basic size. For feature maps whose size is smaller than the basic size, we use bilinear interpolation to adjust to the basic size. In this way, all feature maps in the MSFF module have the same scale size of 38 × 38 on the spatial dimension. (3) Then, the concatenation method is utilized to fuse these feature layers, and the Batch Normalization operation is added to prevent over-fitting, which obtains a feature map with richer semantic information. (4) Finally, 1 × 1 convolution is applied to decrease the number of channels, thereby generating the final feature map. The new feature map combines the low-level feature map and the high-level feature map, which contains rich detailed information and more semantic information. The new feature map is more conducive to small object detection.

### Lightweight receptive field enhancement module

The MobileNetv2-SSD method exploits MobileNetv2 as backbone network to extract features. Although the MobileNetv2 has simple structure and small number of parameters, the extracted features are insufficient. After the backbone network extracts features from the input images, the extracted low-level feature map has small receptive field and the feature expression ability is not strong. Besides, the feature layers with a small receptive field are not conducive to learning small object features. Therefore, an LRFE module is designed to enlarge the receptive field of low-level feature maps. The LRFE not only decreases the complexity of the network but also strengthens the feature discriminability and robustness, thereby improving feature extraction ability.

**Figure 3:**
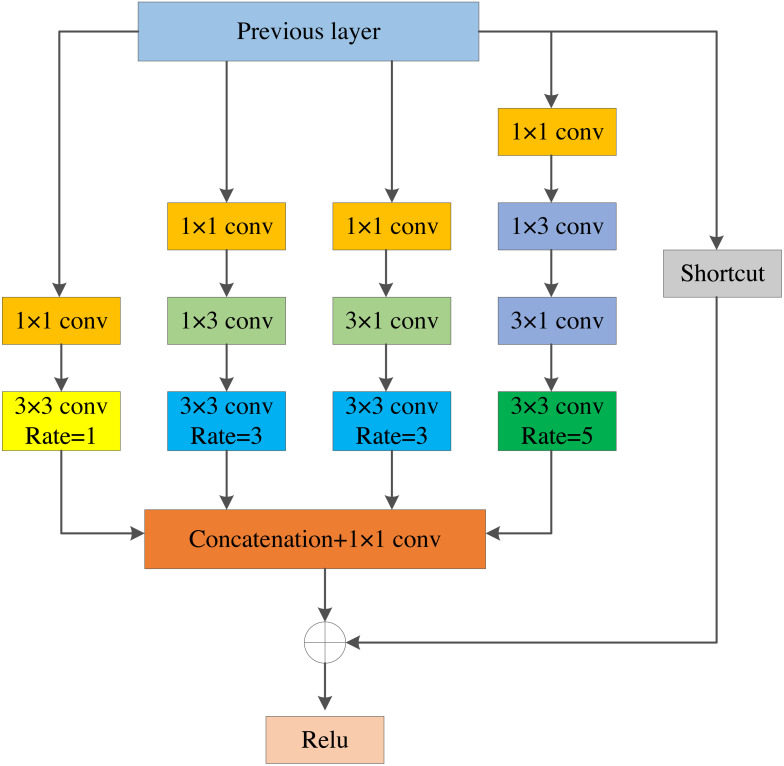
The structure of LRFE module.

Inspired by the Receptive Field Block, we designed the LRFE module. As shown in Fig. 3, it contains four convolution branches and a shortcut branch. To be specific, (1) firstly, 1 × 1 convolution is employed on each convolution branch to reduce the channel dimension of the feature layers. (2) Secondly, the original 3 × 3 conv-layer is replaced by 1 × 3 plus a 3 × 1 conv-layer to make two parallel convolution branches, which reduces the amount of calculation and enhances the features on the width. At the same time, the 5 × 5 conv-layer is substituted by two stacked 1 × 3 and 3 × 1 conv-layers to enhance the features on height. (3) Thirdly, we obtain the feature maps with larger receptive field by using the dilated convolution with dilated rates of 1, 3, 3, and 5. Then, we add the feature maps with the previous layer. (4) Finally, the feature maps containing more contextual information are obtained through the ReLU activation function. The module enlarges the receptive field of feature layers. It is advantageous to use feature layers that contain rich information for fusion in MSFF module.

The LRFE module utilizes multiple branches of different kernels and dilated convolution with different dilated rates to increase the receptive field of feature layers. The feature layers extracted by the backbone network can get a larger receptive field after passing through the LRFE module, which can better preserve the spatial characteristics of the image. The dilated convolutional layer with the dilated rate of 3 can get a receptive field of 9 × 9, and the dilated convolutional layer with the dilated rate of 5 can get a receptive field of 19  × 19. Finally, all branches are connected to generate the final receptive field space array, which effectively increases the receptive field of the feature layer.

### Efficient channel attention module

The MSFF combines large-scale feature layers and small-scale feature layers to generate a new multi-scale feature pyramid for detecting objects. However, these feature layers are independent of each other, and information interaction is incomplete, which is prone to the problems of false detection and missed detection. To solve these problems, we introduce an ECA module into the network architecture. The ECA makes the network ignore the disturbing information and focus on the important features by assigning different weights. The module can efficiently enhance the feature representation ability of the LMSN.

Many studies have demonstrated that the attention mechanism can enhance the overall performance of object detection methods. SE-Net proposed an effective channel attention learning mechanism to learn channel attention for the first time and achieved promising performance. CBAM consisted of two independent sub-modules, namely the channel attention module and the spatial attention module, which performed feature fusion on channel and space, respectively. However, these methods are more complex in structure and mainly focus on improving performance at the expense of speed.

**Figure 4:**
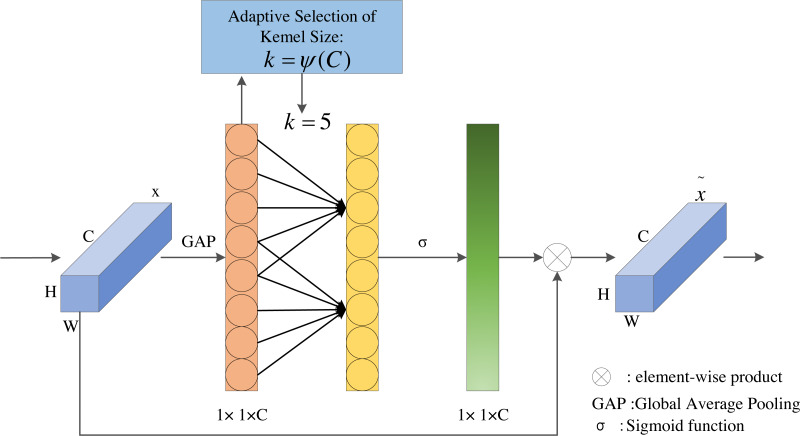
The structure of ECA module.

In order to solve the contradiction between performance and complexity, ECA proposes a proper strategy called local cross-channel interaction without reducing dimensionality and a method to adaptively select kernel size of 1D convolution. The ECA module is a lightweight and efficient attention module, which achieves significant performance improvements with only a few additional parameters. The ECA module is shown in Fig. 4.

As illustrated in Fig. 4, ECA first applies the global average pooling operation on the input feature layers. Next, ECA captures local cross-channel interaction and then obtains the weight of each channel through Sigmoid activation function. Finally, the weights are multiplied by the corresponding elements of the input feature layers to obtain the final output feature layers.

The local cross-channel interaction tactics can be effectively implemented by 1D convolution with kernel size of *k*, the specific calculation is shown in Eq. (2).

(2) \begin{eqnarray*}w=\sigma (C1{D}_{k}(y))\end{eqnarray*}

where *w* is the weights of channels, *σ* is sigmoid function, *C*1*D* represents 1D convolution, *k* is the kernel size of *C*1*D*, and *y* indicates the aggregated feature. Here, the method only involves *k* paraments. When *k* = 3, it can achieve similar effects as SE-var3 while reducing the model complexity.

The ECA module aims to capture local cross-channel interactions appropriately, so the coverage of interaction, *i.e.,* the kernel size of 1D convolution, needs to be determined. Manually optimizing the overage of interaction would cost a lot of computational resources. Therefore, we adopt a method to adaptively determine the convolution kernel *k*, where the size of convolutional kernel *k* is proportional to channel dimension *C* (*i.e.,* there may be a mapping between *k* and *C*). The simplest mapping is the linear function, but the linear mapping has certain limitations. At the same time, we know that the channel dimension is usually an exponential multiple of 2, so we use a base-2 exponential function to represent the nonlinear mapping relationship, as shown in Eq. (3).

(3) \begin{eqnarray*}C=\phi (k)={2}^{(\gamma \ast k-b)}\end{eqnarray*}

The effective feature maps extracted by the LMSN model are 38 × 38 × 512, 19 × 19 × 512, 10 × 10 × 256, 5 × 5 × 256, 3 × 3 × 128, and 1 × 1 × 128, respectively. According to the channel dimensions of different feature maps, the kernel size of 1D convolution should be adaptively selected to determine the coverage of local cross-channel interactions. So the different channels should use different convolutional kernel sizes. The convolutional kernel size *k* can be calculated in Eq. (4).

(4) \begin{eqnarray*}k=\psi (C)={|} \frac{lo{g}_{2(C)}}{\gamma } + \frac{b}{\gamma } {{|}}_{odd}\end{eqnarray*}

where *k* is the size of convolution kernel, *C* is the number of channels, |*t*|_*odd*_ indicates the nearest odd number of *t*, and we set *γ* and *b* to 2 and 1.

## Experiments and Results

In this section, we conduct experiments on PASCAL VOC and RSOD datasets and compare LMSN with state-of-the-art methods. We also design ablation studies to explore the performance of our introduced three modules in LMSN.

### Implementation details

We implement the LMSN model using Pytorch deep learning framework and train it on the Inter i5-11400H, NVIDIA GeForce RTX 3050 GPU. The programming language is Python, and the operating system is Windows 11.

During training, Adam (Yi, Ahn & Ji, 2020) was performed to optimize the network with a momentum of 0.9, the weight decay is 0.0005. The training process is divided into two stages: the freezing stage and the unfreezing stage. It has a total of 100 epochs, and each stage is trained for 50 epochs. In the freezing stage, the initial learning rate is 0.0005, and the batch size is set to 16. In the unfreezing stage, the initial learning rate is set to 0.0001, and the batch size is set to 8. Besides, we use the StepLR mechanism to adjust the learning rate, and the learning rate becomes the original 0.94 for each epoch of training.

The input images are uniformly scaled to 300 × 300 size. Data enhancement methods, such as translational transformations, horizontal flipping, color warping, and random cropping are applied to augment dataset and avoid overfitting.

### Datasets

The PASCAL VOC dataset (Everingham et al., 2010) is an open object detection dataset that includes 20 object categories of different scales and poses. We adopt the trainval set of PASCAL VOC 2007 (Everingham et al., 0000) and PASCAL VOC 2012 (Everingham et al., 0000) (16,551 images) to train the LMSN. The test set of PASCAL VOC 2007 (4,952 images) is used to evaluate the performance.

The RSOD dataset (Long et al., 2017; Xiao et al., 2015) is an open dataset for object detection in remote sensing images that includes aircraft, oiltank, overpass, and playground. Among them, there are 4,993 aircrafts in 466 images, 1,585 oiltanks in 165 images, 180 overpasses in 176 images, and 191 playgrounds in 189 images. During training, the RSOD dataset is divided into trainval set and test set in the ratio of 8:2, and 90% in the trainval set is used for train and 10% for validation.

### Evaluation metrics

We use average precision (AP), mean average precision (mAP), and frame per second (FPS) as evaluation metrics to evaluate the model performance. FPS is the number of frames per second to process the image. AP indicates the area enclosed by the P-R curve and the coordinate axis. The P-R curve is plotted with the recall value as horizontal axis and the precision value as vertical axis. The precision and recall can be defined in Eqs. (5) and (6).

(5) \begin{eqnarray*}Precision= \frac{TP}{TP+FP} \end{eqnarray*}

(6) \begin{eqnarray*}Recall= \frac{TP}{TP+FN} \end{eqnarray*}

where *TP* represents true positive, *FP* represents false positive, *TN* is true negative, and *FN* is false negative. So the AP can be calculated in Eq. (7).

(7) \begin{eqnarray*}AP=\int \nolimits \nolimits _{0}^{1}P(R)dR.\end{eqnarray*}

The mAP is the average value of different kinds of AP. The mAP is computed in Eq. (8).

(8) \begin{eqnarray*}mAP= \frac{A{P}_{1}+A{P}_{2}+\cdots +A{P}_{n}}{n} \end{eqnarray*}

where *n* is the number of all object categories.

### Results and Discussion

#### Results on PASCAL VOC

To demonstrate the effectiveness of LMSN, we compare LMSN and state-of-the-art methods, including Faster R-CNN, YOLOv3, Tiny YOLOv3, MobileNet-YOLOv3, SSD, Tiny SSD, MobileNet-SSD, MobileNetv2-SSD and some improved lightweight methods (Wu et al., 2021; Qi et al., 2020; Zhang, Chen & Xiao, 2021; Cheng et al., 2020b) on the PASCAL VOC dataset. The results are shown in Table 1, where the best results are highlighted in bold.

As we can see from the results, the mAP of LMSN is up to 75.76% and the inference speed is maintained at 61 FPS. Compared to the original MobileNetv2-SSD, the mAP of LMSN is improved by 5.79% while the number of parameters is increased by only 3.9%. Although the detection speed of LMSN is reduced by 36.4% compared with the MobileNetv2-SSD, the proposed method can still meet the requirements of real-time detection. It proves that the LMSN can effectively improve detection accuracy while satisfying real-time detection. Compared to the Faster R-CNN, the mAP of LMSN is improved by 2.56%, the inference speed is increased by 54 FPS. At the same time, the parameters are significantly reduced. It proves that the overall performance of LMSN surpasses the two-stage detection method. Compared with YOLOv3, although the accuracy of LMSN is decreased by 1.34%, the number of parameters is reduced by 89.8%, and detection speed is improved by 26 FPS. Compared with SSD, the mAP of LMSN is increased by 1.46%, the amount of parameters is reduced by 75.8%, and the detection speed is increased by 15 FPS. It proves that LMSN is a lightweight method with promising performance. Compared with the Tiny YOLOv3 and MobileNet-YOLOv3, the mAP is improved by 14.46% and 6.86%, and the number of parameters is reduced by 68.64% and 56.69%, respectively. Compared with the Tiny SSD and MobileNet-SSD, while maintaining a comparable detection speed, the mAP is improved by 14.46% and 3.16%, respectively, which is a significant improvement.

**Table 1:** The experimental results of different methods on PASCAL VOC. Best results are highlighted in bold.

Method	Input	Backbone	mAP (%)	Params (M)	FPS (f/s)
Faster R-CNN	600 × 1000	VGGNet-16	73.2	134.7	7
YOLOv3	320 × 320	Darknet-53	**77.1**	236.3	35
Tiny YOLOv3	416 × 416	Darknet-19	61.3	76.8	**116**
MobileNet-YOLOv3	416 × 416	MobileNet	68.9	55.6	63
SSD	300 × 300	VGGNet-16	74.3	99.7	46
Tiny SSD	300 × 300	SqueezeNet	61.3	**2.3**	–
MobileNet-SSD	300 × 300	MobileNet	72.6	26.3	53
Wu et al. (2021)	300 × 300	MobileNetv2	76.5	26.8	22
Qi et al. (2020)	416 × 416	MobileNet	73.3	23.0	–
Zhang, Chen & Xiao (2021)	416 × 416	MobileNet	75.5	62.4	46
Cheng et al. (2020b)	300 × 300	MobileNetv2	73.8	7.7	97
MobileNetv2-SSD	300 × 300	MobileNetv2	69.97	23.16	96
LMSN	300 × 300	MobileNetv2	75.76	24.08	61

In addition, the performance of LMSN is also better than some improved lightweight algorithms. In particular, the methods proposed by Wu et al. (2021) and Cheng et al. (2020b) both take 300 × 300 input and use MobileNetv2 as the backbone network. Compared with the method proposed by Wu et al. (2021), the mAP of LMSN is reduced by 0.74%, the number of parameters is reduced by 10.1%, and the detection speed is increased by 39 FPS. Although the method proposed by Wu et al. (2021) has high detection accuracy, the detection speed cannot meet the needs of real-time detection. Our LMSN greatly speeds up detection while maintaining high detection accuracy. Compared with the method proposed by Cheng et al. (2020b), the parameters of LMSN are increased by 16.38 M and the detection speed is reduced by 36 FPS, but the mAP is improved by 1.96%. Although the method proposed by Cheng et al. (2020b) has a faster detection speed, the detection accuracy is lower. Our LMSN improves the detection accuracy while satisfying the real-time detection. It can be concluded that LMSN can achieve a good balance in detection speed and accuracy, which is a high-precision and real-time method.

**Figure 5:**
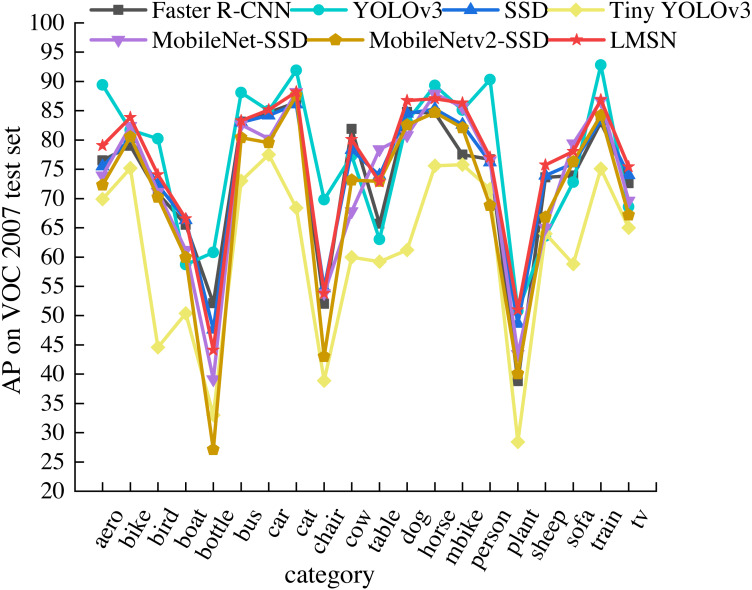
Comparison of AP of 20 categories on the PASCAL VOC 2007 test set.

To detailly analyze the detection performance of LMSN, we compare the detection accuracy of LMSN and state-of-the-art methods in each category of the PASCAL VOC 2007 test set, as shown in Table 2. Among the seven methods listed, we bold the highest AP of single object category. It can be clearly found that the accuracy of LMSN exceeds state-of-the-art methods in most categories. LMSN achieves the best detection results on eight object categories. In other categories, although LMSN does not achieve the best detection results due to the fixed default boxes, AP also surpasses the detection accuracy of many methods. Compared with the Faster RCNN, the detection accuracy of LMSN is improved in 18 categories. The LMSN also surpasses SSD in 18 categories, and only two categories are lower than SSD. Although the detection results of LMSN are only higher than YOLOv3 in 11 categories, the network structure of YOLOv3 is complex and the number of paraments is large, resulting in a slow detection speed. Our LMSN greatly improves the detection speed while ensuring the detection accuracy of multiple categories. In addition, the detection accuracy of LMSN exceeds that of many lightweight methods. The detection accuracy of LMSN is higher than Tiny YOLOv3 in all categories. Compared with MobileNet-SSD, the accuracy of LMSN is only reduced in three categories, while achieving excellent detection results in other categories. Compared with MobileNetv2-SSD, the detection accuracy of LMSN is improved for all object categories. Specifically, for small object classes such as airplane, boat, bottle, chair, plant, sheep, and tv, the detection accuracy is significantly improved by 6.76%, 6.61%, 16.96%, 10.81%, 10.94%, 8.93%, and 8.23%, respectively. Figure 5 shows the visualization results of Table 2. It can be seen intuitively that LMSN is at the highest point in most categories, indicating that LMSN achieves excellent detection performance.

**Table 2:** The AP in each category of the PASCAL VOC 2007 test set. Best results are highlighted in bold.

Method	aero	bike	bird	boat	bottle	bus	car	cat	chair	cow
Faster R-CNN	76.5	79.0	70.9	65.5	52.1	83.1	84.7	86.4	52.0	**81.9**
YOLOv3	**89.4**	81.7	**80.2**	58.7	**60.8**	**88.1**	85.0	**91.9**	**69.8**	77.3
SSD	75.5	80.2	72.3	66.3	47.6	83.0	84.2	86.1	54.7	78.3
Tiny YOLOv3	69.9	75.2	44.6	50.4	33.0	73.0	77.5	68.4	38.9	60.0
MobileNet-SSD	73.9	82.4	71.1	61.2	39.1	82.6	80.2	88.2	53.8	67.8
MobileNetv2-SSD	72.30	80.52	70.19	59.93	27.13	80.40	79.54	87.66	42.98	73.15
LMSN	79.06	**83.48**	74.06	**66.54**	44.09	83.29	**85.14**	88.23	53.79	80.08
Method	table	dog	horse	mobike	person	plant	sheep	sofa	train	tv
Faster R-CNN	65.7	84.8	84.6	77.5	76.7	38.8	73.6	73.9	83.0	72.6
YOLOv3	63.0	83.2	**89.3**	85.1	**90.3**	50.8	63.6	72.8	**92.8**	68.6
SSD	73.9	84.5	85.3	82.6	76.2	48.6	73.9	76.0	83.4	74.0
Tiny YOLOv3	59.2	61.2	75.6	75.8	71.6	28.4	64.0	58.8	75.1	65.0
MobileNet-SSD	**78.4**	80.8	87.9	85.6	76.5	43.47	65.0	**79.4**	86.7	69.6
MobileNetv2-SSD	72.94	82.57	84.70	82.09	68.82	40.05	66.81	76.29	84.21	67.19
LMSN	72.82	**86.74**	87.07	**86.33**	77.05	**50.99**	**75.74**	78.05	86.88	**75.42**

#### Results on RSOD

We also analyzed the detection performance of our proposed LMSN and state-of-the-art methods on RSOD dataset. As shown in Table 3, where the results in bold represent the best performances. From the comparison results of different methods in Table 3, it can be seen that the LMSN surpasses most of the common methods. The mAP of LMSN has reached 89.32% and the inference speed is 64 FPS. Compared to the original MobileNetv2-SSD algorithm, the mAP of LMSN is improved by 11.14%, and the detection speed is decreased by 37 FPS. Real-time is still possible despite the reduced detection speed. It proves that our LMSN obviously improves the model accuracy while taking into account the detection speed. At the same time, the accuracy of LMSN in each category also surpasses the MobileNetv2-SSD method, especially the AP of aircraft and oiltank are improved by 22.48% and 8.51%, respectively. Compared to the Faster R-CNN, the mAP of our model is improved by 1.56%, and the detection speed is improved by 56 FPS. Compared with the YOLOv3, although the accuracy of LMSN is reduced by 1.95%, the detection speed is improved by 35 FPS. Compared to the YOLOv2, the mAP of LMSN is increased by 20.07%, and the detection speed is increased by 28 FPS, which proves that the LMSN surpasses the YOLOv2 in both accuracy and speed. The detection accuracy of LMSN is 1.96% higher than SSD. The detection speed of LMSN is 16 FPS higher than SSD. Compared with the lightweight methods, such as Tiny YOLOv3 and Tiny YOLOv4, the mAP of LMSN is improved by 30.86% and 2.85%, respectively, while keeping a comparable detection speed. These results demonstrate the effectiveness of LMSN.

**Table 3:** The detection performance of different methods on RSOD dataset. Best results are highlighted in bold.

Method	Backbone	AP (%)				mAP (%)	FPS (f/s)
		Aircraft	Oiltank	Overpass	Playground
Faster R-CNN	VGG-16	85.85	86.67	88.15	90.35	87.76	8
YOLOv3	DarkNet53	84.80	99.10	81.20	100.00	**91.27**	29
YOLOv2	Darknet19	62.35	67.74	68.38	78.51	69.25	36
SSD	VGG-16	57.05	98.89	93.51	100.00	87.36	48
RetinaNet	ResNet-101	80.57	96.97	96.69	90.25	91.19	–
Tiny YOLOv3	DarkNet19	54.14	56.21	59.28	64.20	58.46	69
Tiny YOLOv4	CSPdarknet53-tiny	66.47	99.42	80.68	99.31	86.47	54
Wu et al. (2021)	MobileNetv2	73.06	98.20	71.86	100.00	85.78	68
Cheng et al. (2020b)	MobileNetv2	66.02	89.69	88.52	100.00	86.06	42
MobileNetv2-SSD	MobileNetv2	50.51	89.94	72.27	100.00	78.18	**101**
LMSN	MobileNetv2	72.99	98.45	85.81	100.00	89.32	64

In addition, we compared the LMSN with the methods proposed by Wu et al. (2021) and Cheng et al. (2020b). These methods are also lightweight object detection algorithms using MobileNetv2 as the backbone network. Compared with the method proposed by Wu et al. (2021), the mAP of LMSN is improved by 3.54%, while the detection speed is only reduced by 4 FPS. Although the detection speed of LMSN is slightly reduced, the detection accuracy is greatly improved. The LMSN achieves a better balance in detection accuracy and speed. Compared with the method proposed by Cheng et al. (2020b), the mAP of LMSN is increased by 3.26%, and detection speed is increased by 22 FPS. It can be seen that the LMSN surpasses the method proposed by Cheng et al. (2020b) both in accuracy and speed. In conclusion, compared with these improved lightweight algorithms, our proposed LMSN achieves better detection performance on RSOD dataset and can detect small objects more efficiently.

**Figure 6:**
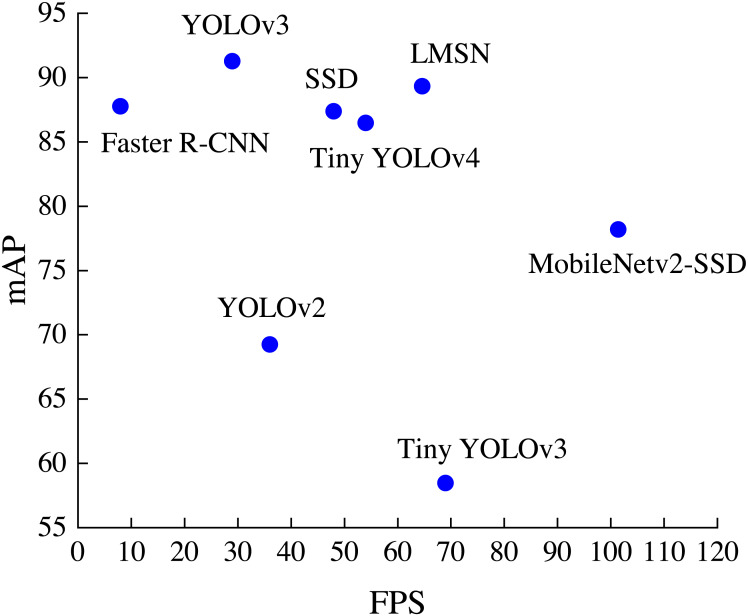
The distribution of accuracy and speed with different methods.

The distribution of detection accuracy and detection speed of different methods on the RSOD dataset is shown in Fig. 6. It can be found that LMSN surpasses Faster R-CNN, YOLOv2, YOLOv3, SSD and Tiny YOLOv4 in speed, and is better than Faster R-CNN, SSD, YOLOv2, Tiny YOLOv3, Tiny YOLOv4 and MobileNetv2-SSD in accuracy. Overall, our LMSN achieves excellent detection performance in both detection accuracy and detection speed.

### Five-fold cross validation experiments

To evaluate the generalization and stability of the method, we conduct five-fold cross-validation experiments on RSOD dataset. The basic steps are as follows: (1) Divide the RSOD dataset into five equal subsets. (2) Use the first subset as the test set, and the other four subsets are combined as the train set. (3) Train the model and calculate the mAP of the model under the test set. (4) Repeat steps (2)-(3), taking the second to fifth subsets as the test set in turn. (5) Calculate the average value of mAP obtained from five experiments. By conducting the five-fold cross-validation experiments, we obtained the mAP and its average value of five experiments of MobileNetv2-SSD and LMSN, respectively, as shown in Table 4.

As can be seen from Table 4, in the five experiments of MobileNetv2-SSD, although the result of Test4 is low, the results of the other four experiments are not much different, which proves that the model is relatively stable. The five results of our LMSN are relatively close, which can effectively illustrate the stability of our proposed model. At the same time, it can be seen from Table 4 that the average mAP of MobileNetv2-SSD is 78.11%, and the average mAP of LMSN reaches 88.47%. Compared with the MobileNetv2-SSD, the mAP of LMSN is improved by 10.36%, which effectively proves the effectiveness of LMSN.

### Ablation studies

To qualitatively analyze the effectiveness of each module in LMSN, we design ablation studies on the RSOD dataset. Experiment A is the MobileNetv2-SSD algorithm and Experiment B to E are methods with our proposed module, where ‘√’ indicates that the proposed module is applied and ‘×’ indicates that the proposed module is not used. The results of ablation studies are shown in Table 5.

**Table 4:** The results of five-fold cross-validation experiments on RSOD dataset.

Method	Test1 (%)	Test2 (%)	Test3 (%)	Test4 (%)	Test5 (%)	Average (%)
MobileNetv2-SSD	78.65	79.33	79.10	75.22	78.27	78.11
LMSN	88.87	88.68	89.24	87.06	88.52	88.47

**Table 5:** The results of ablation studies on the RSOD dataset.

Groups	MSFF	LRFE	ECA	mAP (%)	FPS (f/s)
Experiment A	×	×	×	78.18	101.42
Experiment B	√	×	×	85.78	96.93
Experiment C	√	×	√	87.75	84.19
Experiment D	√	√	×	87.08	68.23
Experiment E	√	√	√	89.32	64.64

Experiment A is the MobileNetv2-SSD object detection algorithm. The mAP is 78.18% and FPS is 101.42. MobileNetv2-SSD achieves good detection results on RSOD dataset, but it is inconvenient to detect some small targets with low resolution and dense distribution. There are problems of missed detection and false detection.

Experiment B adds MSFF module based on MobileNetv2-SSD. The mAP is increased from 78.18% to 85.78% and FPS is 96.93, which proves that MSFF can effectively integrate the detailed information of shallow feature layers and the semantics information of deep feature layers. Experiment B achieves a better detection performance and detects more small objects. It illustrates that the MSFF module obtains the low-level feature layers with both detailed features and rich semantic representation, which improves the detection accuracy for small objects.

Experiment C combines MSFF module and ECA module, which greatly improves the detection performance with a slight decrease in detection speed. The mAP is increased from 85.78% to 87.75% and FPS is 84.19. It proves that the ECA can strengthen the association between each feature layer, pay more attention to the key features, and strengthen the feature representation capabilities. Experiment C heightens the detection accuracy of small targets and reduces the situation of missed detection of small-scale objects.

Experiment D adds LRFE module to the MSFF module. The mAP is increased from 85.78% to 87.08% and FPS is 68.23, indicating that the LRFE can effectively enlarge the receptive field size of feature maps and enhance the feature extraction ability. Compared with Experiment B, Experiment D further heightens the detection effort of small-scale targets with low-resolution and improves the robustness of the network. It also shows that the combination of MSFF module and LRFE module can achieve a better detection performance.

Experiment E is the LMSN model proposed in this article. LMSN contains our proposed three modules. The mAP is increased to 89.32% and FPS is 64.64. Compared with the original MobileNetv2-SSD, the mAP increases by 11.14% while reducing the detection speed only slightly. Compared to other experiments, Experiment E achieves the best detection performance for small objects with low resolution and dense distribution. It can be concluded that our designed three modules can effectively strengthen the detection accuracy while maintaining the detection speed.

## Conclusion

This article proposed a lightweight multi-scale network, called LMSN, which is equipped with three effective modules. To solve the problem that small targets are difficult to identify in complex scenes, an MSFF module was designed to strengthen the semantic expressiveness of low-level feature maps. Then, an LRFE module was added to enlarge the receptive field of feature maps, which enhanced the feature extraction capability of the network. Additionally, the ECA module was introduced to suppress irrelevant background information and enhance the feature representation ability. The experimental results on PASCAL VOC and RSOD datasets demonstrated that LMSN outperforms most popular methods. It achieved superior detection performance with competitive inference speed. However, there is still room for improvement in small object detection, especially the small targets with heavy occlusion. The proposed network structure will be further improved in future work.

## Additional Information and Declarations

### Data Availability

The following information was supplied regarding data availability: The code is available at GitHub: https://github.com/Lbx2020/LMSN; Lbx2020. (2022). Lbx2020/LMSN: First release of my LMSN (v1.0.0). Zenodo. https://doi.org/10.5281/zenodo.7145670. The data is available at: - PASCAL VOC dataset: http://host.robots.ox.ac.uk/pascal/VOC/. Everingham et al. (2010). The pascal visual object classes (voc) challenge. (https://doi.org/10.1007/s11263-009-0275-4). - PASCAL VOC 2007 dataset: http://host.robots.ox.ac.uk/pascal/VOC/voc2007/index.html. Everingham et al. (0000). - The PASCAL Visual Object Classes Challenge 2007 (VOC2007) Results. http://www.pascalnetwork.org/challenges/VOC/voc2007/workshop/index.html. - PASCAL VOC 2012 dataset: http://host.robots.ox.ac.uk/pascal/VOC/voc2012/index.html. Everingham et al. (0000). The PASCAL Visual Object Classes Challenge 2012 (VOC2012) Results. http://www.pascalnetwork.org/challenges/VOC/voc2012/workshop/index.html. - RSOD dataset: https://github.com/RSIA-LIESMARS-WHU/RSOD-Dataset-#rsod-dataset. Wuhan University Team, Long et al. (2017). Accurate object localization in remote sensing images based on convolutional neural networks. (https://doi.org/10.1109/TGRS.2016.2645610). Xiao et al. (2015). Elliptic fourier transformation-based histograms of oriented gradients for rotationally invariant object detection in remote-sensing images. (https://doi.org/10.1080/01431161.2014.999881).
